# Associating *Mycobacterium tuberculosis* genomic variants with minimum inhibitory concentrations to delamanid and pretomanid

**DOI:** 10.1371/journal.pone.0359717

**Published:** 2026-10-05

**Authors:** Ana Tsutsunava, Nestani Tukvadze, Zaza Avaliani, Galo A. Goig, Levan Jugheli, Sebastien Gagneux, Russell R. Kempker, Nino Maghradze

**Affiliations:** 1 National Center for Lung Health (NCLH), Tbilisi, Georgia; 2 Swiss Tropical and Public Health Institute (Swiss TPH), Allschwil, Switzerland; 3 University of Basel, Basel, Switzerland; 4 European University, Tbilisi, Georgia; 5 Division of Infectious Diseases, Department of Medicine, Emory University School of Medicine, Atlanta, Georgia, United States of America; Lady Hardinge Medical College, INDIA

## Abstract

Emerging resistance to delamanid (DLM) and pretomanid (Pa) threatens drug-resistant tuberculosis (DR-TB) treatment outcomes. The lack of rapid molecular diagnostics, due to limited knowledge of resistance-conferring mutations, along with standardization challenges for critical concentrations (CC) in phenotypic drug susceptibility testing (pDST) hinder resistance detection. Here, we explore the correlation between minimum inhibitory concentrations (MICs) and genomic variants of DLM and Pa resistance in *Mycobacterium tuberculosis* (*Mtb*)*.* Seventeen *Mtb* isolates phenotypically resistant to DLM detected through routine pDST using the Mycobacterial Growth Indicator Tube (MGIT) system from 2021–2024 were selected from the National Reference Laboratory biobank in Tbilisi, Georgia. DLM/Pa MICs were determined using the Resazurin Microtiter Assay (REMA) with a DLM concentration range of 0.008–2 µg/ml, and a broader range of 0.125–32 µg/ml was utilized for Pa. To identify mutations associated with DLM/Pa resistance, isolates were subjected to whole genome sequencing (WGS). Of the seventeen *Mtb* isolates phenotypically resistant to DLM, eight (47%) manifested a MIC value below the DLM CC (0.06 µg/ml), with only one carrying a mutation in a gene associated with DLM/Pa resistance (*fbiC*). Among the remaining nine isolates with MICs above the CC, the premature stop codon mutation W88* in *ddn* gene predominated, present in five cases and associated with elevated DLM and Pa MICs (0.125–2 µg/ml and 2–8 µg/ml, respectively). Three isolates (19%) harbored novel, previously undescribed genetic variants, all within genes previously linked to resistance DLM/Pa (G35fs in *ddn*, Q317R and Q279H in *fgd1*, and C565W in *fbiC*). Our results support DLM and Pa resistance association with W88* and W678G mutations in *ddn* and *fbiC* genes respectively, currently graded as “uncertain significance” in the WHO catalogue of *Mtb* drug resistance-conferring mutations. Additionally, we observed previously undescribed mutations exclusively within genes linked to DLM/Pa susceptibility, prompting further investigation of their role in resistance.

## Introduction

Surpassing COVID-19, with an estimated 1.23 million deaths in 2024, TB reclaimed to be the world’s leading cause of death from a single infectious agent [1]. While the introduction of novel medications against drug-resistant TB, such as bedaquiline (BDQ), delamanid (DLM) and pretomanid (Pa) have increased treatment success rates, the global increase in emerging resistance towards these medications is concerning and threatens their long term-efficacy [2–4].

DLM, approved by the European Medicines Agency (EMA) in 2014 is a part of individualized treatment regimens recommended for all persons with rifampicin resistant and multi-drug resistant (RR/MDR) TB for all age groups. Classified as group C medication, it is a major alternative drug in case of resistance or intolerance towards group A and B agents, moreover it is included in a newly recommended regimen for RR/MDR-TB alongside bedaquiline, linezolid, levofloxacin, and clofazimine (6-month BDLLfxC) [5,6]. Pa – the latest addition to the anti-TB drug arsenal, was approved by the United States Food and Drug Administration (FDA) in 2019 for use in the BPaL regimen, which combines Pa with BDQ and linezolid, later recommended by WHO as regimen of first choice with or without moxifloxacin [5–7]*.*

Both compounds – DLM and Pa, are prodrugs and share the same activation pathway involving the mycobacterial F420-dependent nitro-reductase encoded by the *ddn* (*Rv3547*) gene [8]. Co-factor F420 is synthesized and activated by a group of enzymes encoded by the genes *fgd1* (Rv0407), *fbiA* (Rv3261), *fbiB* (Rv3262), *fbiC* (Rv1173) and *fbiD* (Rv2983) [9]. With a similar mechanism of action, these two drugs inhibit the mycobacterial cell wall synthesis, leading to cell death. Various mutations in these genes have been associated with an increased MICs to DLM and Pa [10] additionally confirming the presence of cross-resistance between these two medications [11,12]. Although the exact molecular mechanisms of action of DLM and PA are not yet fully understood, recent studies have described the essential enzyme in cell wall biosynthesis -decaprenylphosphoryl-2-keto-β-D-erythro-pentose reductase (*DprE2*), as a shared target of both drugs implicating possible role in resistance [13].

To preserve novel drugs and regimens, precise and prompt detection of resistance to these medications is crucial. The World Health Organization (WHO) recommends performing drug susceptibility testing (DST) to detect resistance to DLM and Pa. For DLM, a critical concentration (CC) of 0.06 μg/ml is currently recommended for MGIT-based testing [14]. However, interpreting Pa susceptibility remains more complex. Early studies suggested a single critical concentration of 1 μg/ml for pretomanid based on available MIC data [15]. Nevertheless, subsequent studies showed that MIC values vary by *Mycobacterium tuberculosis* (*Mtb)* lineage, with lineage 1 strains generally exhibiting higher MICs [15–17]. As a result, WHO now recommends testing Pa at two concentrations (0.5 and 2 μg/ml) in MGIT. Growth at 2 μg/ml is used to define resistance, while results at lower concentrations should be interpreted with caution [18]. In contrast, the European Committee on Antimicrobial Susceptibility Testing (EUCAST), which establishes clinical breakpoints on behalf of EMA, defines the breakpoint for Pa at 2 μg/ml using the MGIT system [19].

The WHO mutation catalogue (2nd edition) [20], based on highly conservative selection criteria, describes 24 mutations associated with DLM resistance classified as Group 2 - “associated with resistance interim”. In addition, the WHO recommends that any loss-of-function mutation in the six resistance-associated genes (*ddn*, *fgd1*, *fbiA, fbiB*, *fbiC*, and *fbiD*) to be interpreted similarly as a Group 2 mutation. Notably, six such loss-of-function mutations are listed in the catalogue annex [20]. The catalogue further includes resistance-associated mutations for Pa, including shared mutations such as *ddn* W88*, which are classified as Group 2 for both nitroimidazoles.

In addition, uncertainty around the associations of *Mtb* genetic variants with phenotypic resistance to nitroimidazoles highlights a key gap that needs to be addressed. In this regard, the combination of the comprehensive MIC data, pDST and whole genome sequencing (WGS) can help identify the genetic determinants of resistance, and in developing rapid diagnostics [21]. We aimed to link DLM phenotypic susceptibility (BACTEC MGIT 960 system) and DLM/Pa MIC results (REMA assay) with WGS data.

## Materials and methods

### Ethics

This study was conducted with approval from the Institutional Review Board (IRB) of the National Center for Lung Health (NCLH; formerly, National Center for Tuberculosis and Lung Disease) in Tbilisi, Georgia. While designed and executed independently, it utilized biobanked isolates and genomic data generated through a nationwide genomic epidemiology project funded by the European Research Council (ERC), in collaboration with the Swiss Tropical and Public Health Institute (Swiss TPH), for which the necessary ethical approvals had already been obtained in both Georgia and Switzerland (Ethikkommission Nordwest- und Zentralschweiz). Given the population-wide scope of the genomic study, the Ministry of Labor, Health and Social Affairs of Georgia, along with the local IRB, granted a waiver of written informed consent for both prospective and retrospective TB cases. All clinical and sample-related data used in this study were pseudonymised and the authors had no access to any personally identifiable information at any stage during or after data collection.

### *Mtb* isolate selection

We identified *Mtb* isolates with phenotypic resistance to DLM through the National TB Program (NTP) laboratory database that encompasses the TB diagnostic test results from across the country. Routine DST for DLM was implemented by the NCLH in February 11, 2021 using the BACTEC MGIT 960 system with a CC of 0.06 µg/ml, as recommended by WHO [14]. All routine DLM pDST was performed at the NRL, NCLH, ensuring methodological consistency across the study period, with continuously maintained regular external quality assessment using blinded strains, through participation in the WHO Supranational Reference Laboratory Network (SRLN). Baseline *Mtb* isolates were selected from TB cases diagnosed between February 11, 2021 and July 31, 2024. Routine DLM phenotypic DST was performed in patients with RR-TB as part of national programmatic practice. Clinical and bacteriological metadata, including previous TB history, TB profile, treatment regimen, and treatment outcomes, were obtained from the NTP surveillance electronic database of the NCLH.

### MIC assay

All available baseline DLM resistant *Mtb* isolates retrieved from the NCLH Biobank, were sub-cultured on Löwenstein-Jensen (LJ) medium, and subsequently subjected to MIC testing against DLM and Pa using REMA [22]. Stock solutions of drugs prepared in DMSO were frozen at −20°C in aliquots for up to 6 months and discarded after single thawing for the assay preparation. Plates containing serial dilutions of DLM or Pa were prepared in advance. Before inoculation, bacterial suspensions were adjusted in saline–Tween broth to a turbidity equivalent to McFarland 0.5 using a nephelometer, then added to the plates. The concentration ranges were as follows: 2/1/0.5/0.25/0.125/0.06/0.03/0.016/0.008 μg/ml for DLM and 32/16/8/4/2/1/0.5/0.25/0.125 μg/ml for Pa. A broader concentration range was explored for Pa due to the absence of routine pDST data. Isolates were tested in batches under standardized conditions. Each batch included well characterized laboratory reference susceptible strain H37Rv as a quality control, drug-free wells as positive growth controls, and wells without inoculum as negative controls. H37Rv MICs remained consistently ≤0.008 μg/mL for DLM and ≤0.125 μg/mL for Pa. Because these values corresponded to the lowest concentrations tested, exact MIC endpoints could not be determined.

Alamar blue was added to the plates, which were incubated at 37 ºC. After 21 days, the MIC was defined as the lowest drug concentration where the growth was observed in triplicate wells, as determined by visual color change. Growth and contamination were monitored throughout the incubation period, and results were recorded accordingly.

### Genomic data

DNA from study isolates were extracted using phenol-CTAB method, measured on Nanodrop spectrophotometer for assessing quantity and purity and sent for whole genome sequencing at the genomic core facility of the University of Basel and the Department of Biosystems Science and Engineering at ETHZ in Basel, Switzerland. For sequencing the NEBNext Ultra II FS DNA Library Prep Kit with an Illumina NovaSeq 6000 and an Illumina HiSeq 2500 platform were used.

We identified nonsynonymous mutations, frameshifts, indels, or nonsense mutations in the DLM/Pa resistance-associated genes *ddn, fgd1, fbiA, fbiB, fbiC,* and *fbiD.* In our study we also explored mutations within *dprE2* as a shared target for both drugs. Sequencing reads from samples passing quality criteria were taxonomically classified with Kraken [23] and non-MTBC reads were removed. The resulting reads were aligned to the genome of the inferred MTBC ancestor (10.5281/zenodo.3497110), and variants were called using the microbial mode of GATK Mutect2. Variants in repetitive regions such as PE, PPE, and PGRS genes were removed. Resistance profiles for each isolate were assigned based on the presence of mutations in resistance-associated genes in accordance with the latest WHO catalogue and definitions.

Phylogenetic analysis was performed to explore the evolutionary relationships among the *Mtb* isolates and emergence of the mutations, assess whether specific resistance-associated variants were lineage-specific, providing insights into the potential spread and genetic context of DLM/Pa resistance. To infer a maximum-likelihood phylogeny we built a multiple alignment of all non-redundant single-nucleotide polymorphisms (SNPs). In this alignment, we only considered SNPs with a minimum intra-sample allele frequency of 90%. The phylogeny was inferred using IQ-TREE 2 [24], specifying the general time-reversible model of sequence evolution, indicating the number of invariant sites of each nucleotide, and rooted using *M. canettii* as an outgroup.

## Results

### Isolate selection

During the study period (2021–2024), a total of 469 patients were diagnosed with RR-TB by Xpert MTB/RIF Ultra countrywide. Routine DLM pDST was performed for 425 (90.6%) patients as part of the NTP, whereas DLM pDST was unavailable for 44 (9.4%) patients (overlap with validation of the DLM pDST workflow, lack of a clinician request, or drug unavailability). Of the 425 patients tested, 32 (7.5%) isolates were initially classified as DLM resistant. Although repeat testing is not routinely performed, it may be undertaken based on the overall drug resistance profile and clinical context. All 32 isolates underwent repeat MGIT testing, after which 11 isolates were reclassified as susceptible and excluded from further analysis. This resulted in 21 isolates (4.9%) of the total tested being reported as DLM-resistant. Among these, one isolate failed regrowth and three lacked WGS data, leaving 17 isolates available for further characterization using REMA MIC and WGS (Fig 1).

**Fig 1:**
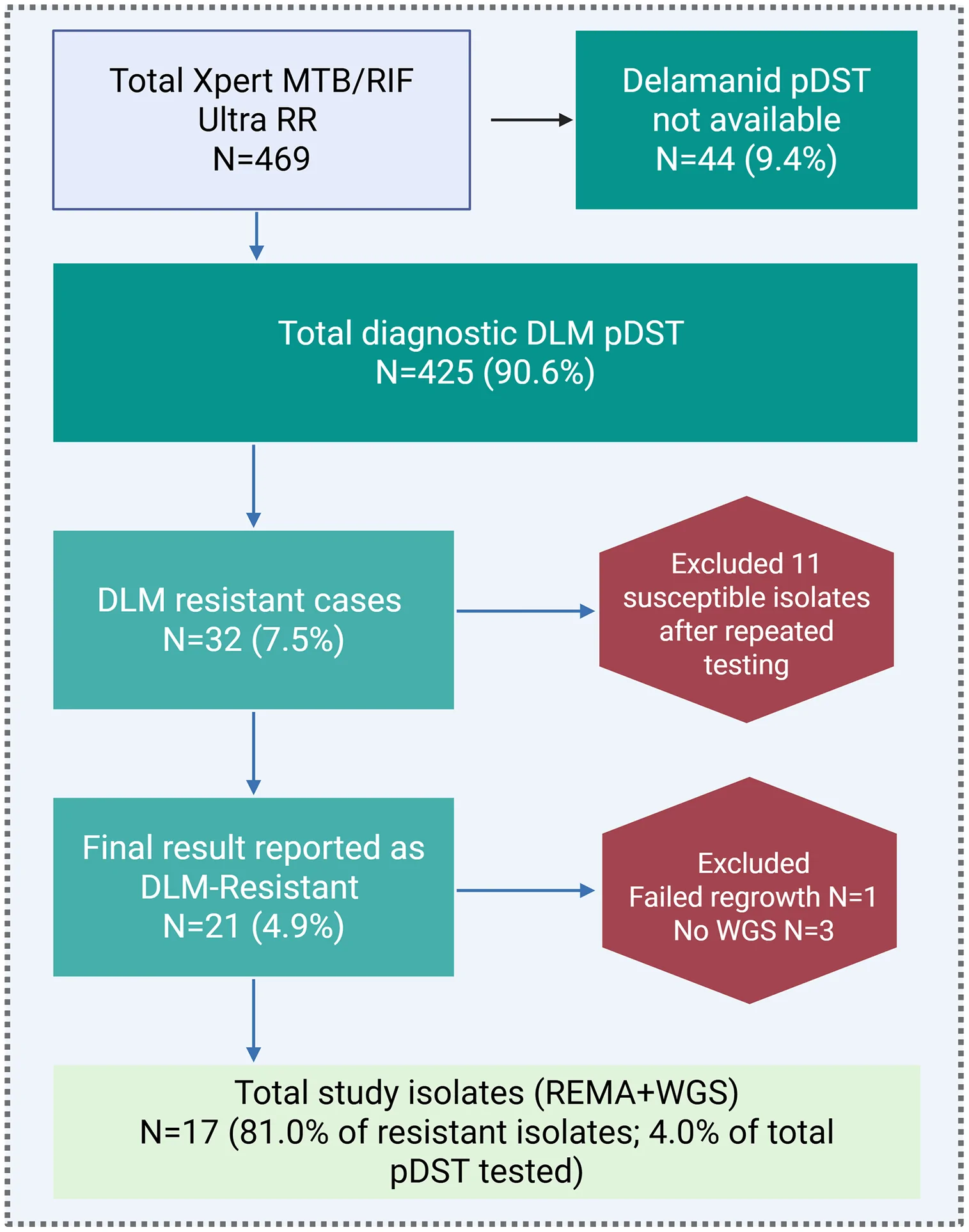
Flow diagram of *Mtb* isolate selection. Flow diagram of baseline *Mycobacterium tuberculosis* isolate selection from rifampicin-resistant tuberculosis (RR-TB) patients, including routine delamanid (DLM) phenotypic drug susceptibility testing (pDST), repeat testing of initially resistant isolates, and the final study isolates analyzed by REMA minimum inhibitory concentration (MIC) testing and whole genome sequencing (WGS).

### MIC distribution

REMA testing revealed that 8 (47%) out of the 17 isolates had a DLM MIC value below the CC (0.06 μg/ml), indicating susceptibility. Seven of these isolates exhibited growth only in drug-free wells, with MICs interpreted as ≤0.008 μg/ml for DLM and ≤0.125 μg/ml for Pa, representing the lowest concentrations tested in the REMA assay. All isolates phenotypically susceptible to DLM were also susceptible to Pa, except for one isolate consistently showing a DLM MIC of 0.016 μg/ml, confirmed through repeated testing, that corresponds to the next lowest concentration after 0.008 μg/ml for DLM, with a Pa MIC of 2 μg/ml.

The MIC distribution for the remaining 9 isolates was as follows: 6 isolates (67%) exhibited a MIC of 2 μg /ml for DLM, 2 isolates (22%) had a MIC of 0.125 μg /ml and 1 isolate (11%) had MIC of 0.25 μg /ml. All 9 phenotypically resistant DLM strains (by REMA) demonstrated higher Pa MIC values, ranging from 2 to 8 μg /ml.

### Genomic data

Among the 17 isolates analyzed, 9 (53%) harbored mutations in genes associated with DLM/Pa resistance according to the WHO catalogue [20], specifically *ddn*, *fgd1*, *fbiA*, *fbiB*, *fbiC*, and *fbiD*. Of these, only one isolate was not confirmed as phenotypically resistant by REMA (Table 1). Within this group, ddn mutations predominated (67%), especially the formerly described [9,17,20,25] premature stop codon mutation W88* (56%) and occurred in isolates with high MICs for DLM (0.125–2 μg/ml) and Pa (4–8 μg/ml). While *fbiA, fbiB*, *fbiD* and *dprE2* mutants were not present in our dataset, 3 isolates (33%) carried mutations in the *fbiC* gene, specifically *FbiC* C565W and FbiC W678G. Regarding the *fgd1* gene, we detected mutations (Q317R, Q279H) in 2 isolates (22%). Out of the detected 6 genomic variants with allele frequency >10%, 4 variants G35fs (33.3%) in *ddn*, Q317R (22.1%) and Q279H (15.5%) in *fgd1*, and C565W detected in two isolates with allele frequencies of 42.7% and 97% in *fbiC* were exclusively in our sample set and not described elsewhere including WHO mutation catalogue (Table 1). Conversely, among the remaining 8 isolates with DLM MIC values below CC, all showed wild-type (WT) alleles in DLM/Pa resistance-conferring genes, apart from one isolate with *fbiC* W678G (96.7%) mutation. Two isolates carrying the *atpE* Ile66Met mutation differed by ≤5 SNPs and belonged to the same sublineage, a genomic relationship compatible with a close epidemiological link.

**Table 1:** Delamanid (DLM) and pretomanid (Pa) minimum inhibitory concentrations (MICs), genomic variants, lineage, and clinical characteristics of 17 *Mycobacterium tuberculosis* isolates included in the study.

G-Number	MIC (μg/ml)		Genomic Data							Epidata		
	*DLM*	*Pa*	*ddn*	*fgd1*	*fbiA*	*fbiB*	*fbiC*	*fbiD*	*dprE2*	*Lineage*	*Treatment regimen*	Outcome***
G724028	0.25	8	G35fs (33.3%)**	Q317R (22.1%)	WT	WT	C565W (42.7%)	WT	WT	2.2.1 Europe_Russia_W148 outbreak	Pto Cs AMX-CLV CFZ	Treatmentfailed
G703244	2	4	W88* (99.8%)	WT	WT	WT	WT	WT	WT	2.2.1 Europe_Russia_W148 outbreak	LFX Cs CFZ Lzd Bdq	Cured
G703277	0.125	8	W88* (99.8%)	WT	WT	WT	WT	WT	WT	2.2.1 Europe_Russia_W148 outbreak	LFX Cs CFZ Lzd Bdq	Cured
G702636	0.125	8	W88* (99.8%)	Q279H (15.5%)	WT	WT	WT	WT	WT	2.2.1 Europe_Russia_W148 outbreak	Lzd Bdq	Treatmentfailed
G702708	2	4	W88* (99.4%)	WT	WT	WT	WT	WT	WT	2.2.1 Europe_Russia_W148 outbreak	LFX Cs CFZ Lzd Bdq	Lost to follow up
G742413	≤0.008	≤0.125	WT	WT	WT	WT	**W678G** (96.7%)	WT	WT	4.3.3	Lzd Bdq	Cured
G724056	2	2	WT	WT	WT	WT	C565W (97.0%)	WT	WT	2.2.1 Europe_Russia_W148 outbreak	Pto Cs PAS AMX-CLV CFZ	Not evaluated
G724063	2	2	WT	WT	WT	WT	**W678G** (99.4%)	WT	WT	4.3.3	PAS AMX-CLV Lzd	Death
G748744	2	8	W88* (99.4%)	WT	WT	WT	WT	WT	WT	2.2.1 Europe_Russia_W148 outbreak	LFX Cs CFZ Lzd Bdq	Cured
G703226	≤0.008	≤0.125	WT	WT	WT	WT	WT	WT	WT	2.2.1 Central_Asia	LFX Cs CFZ Lzd Bdq	Cured
G703239	≤0.008	≤0.125	WT	WT	WT	WT	WT	WT	WT	2.2.1	LFX Cs CFZ Lzd Bdq	Cured
G702633	≤0.008	≤0.125	WT	WT	WT	WT	WT	WT	WT	2.2.1 Central_Asia	LFX Cs CFZ Lzd Bdq	Cured
G703262	≤0.008	≤0.125	WT	WT	WT	WT	WT	WT	WT	2.2.1 Central_Asia	Pto Cs Bdq Dld	Treatmentfailed
G702690	≤0.008	≤0.125	WT	WT	WT	WT	WT	WT	WT	2.2.1 Europe_Russia_W148 outbreak	LFX Cs CFZ Lzd Bdq	Lost to follow up
G702713	0.0156	2	WT	WT	WT	WT	WT	WT	WT	2.2.1 Europe_Russia_W148 outbreak	Lzd Bdq	Lost to follow up
G702673	≤0.008	≤0.125	WT	WT	WT	WT	WT	WT	WT	2.2.1 Europe_Russia_W148 outbreak	LFX Cs CFZ Lzd Bdq Dlm	Lost to follow up
G742988	2	4	WT	WT	WT	WT	WT	WT	WT	4.3.3	Lzd Bdq	Not evaluated

Table 1 represents the data of 17 phenotypically resistant isolates to DLM (BACTEC MGIT 960system) with corresponding MIC results for DLM/Pa via REMA assay, and mutations in resistance conferring genes: ddn (Rv3547), fgd1 (Rv0407), fbiA (Rv3261), fbiB (Rv3262), fbiC (Rv1173), fbiD (Rv2983) in addition with dprE2 (Rv3791); W88*- premature stop codon; ** Variant allele frequency; Mutations in bold are defined as uncertain significance to resistance in WHO mutation catalogue (2nd edition). ***Treatment outcomes are defined according to World Health Organization (WHO) guidelines.

In our dataset, 14 isolates (82%) belonged to lineage 2 and three isolates (18%) to lineage 4. Among the eight isolates classified as resistant by MGIT but with MIC values below the critical concentration by REMA, seven belonged to lineage 2 and one to lineage 4, reflecting the overall lineage distribution of the dataset and indicating no lineage-specific clustering of discordant results. Phylogenetic analysis showed the distribution of resistance-associated mutations across *Mtb* lineages, highlighting their predominance in the L2.2.1 sub-lineage, whereas premature W88 stop codon was detected exclusively in the W148 outbreak cluster (Fig 2). Isolate sequencing data have been deposited in the European Nucleotide Archive under the study project PRJEB39561 and accession numbers are provided in supplementary Table 1 (S1 Table).

**Fig 2:**
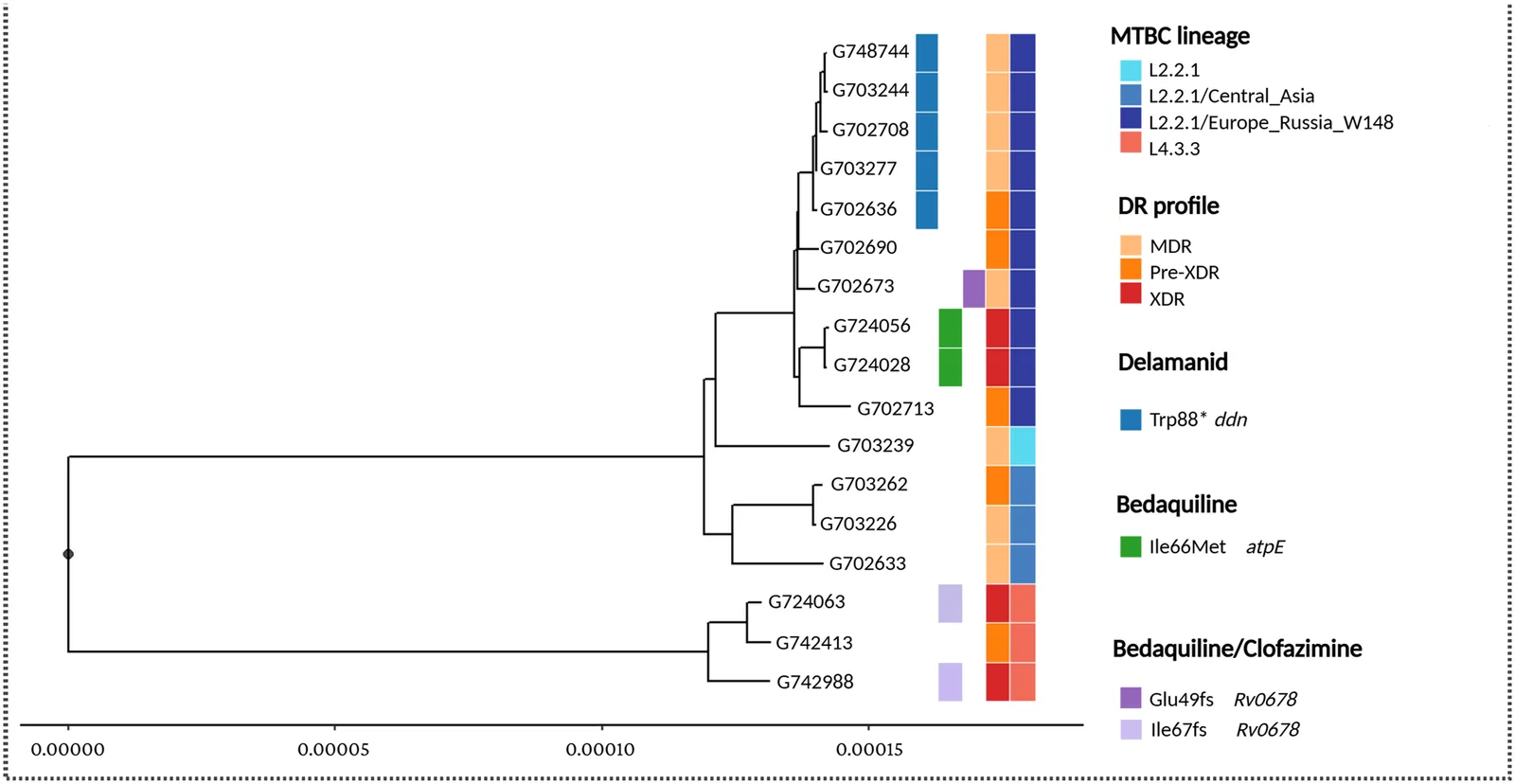
Maximum likelihood phylogeny of the study isolates with mutations detected in genes associated with resistance to DLM/Pa (in blue), bedaquiline (in green), bedaquiline and clofazimine (in purple), resistance profile and MTBC lineage.

### Epidemiological metadata

Out of 17 samples analyzed, 12 (71%) were from patients with a prior TB episode, including 4 with DLM treatment history (all with unsuccessful treatment outcomes), 5 without, and 3 with missing history on DLM treatment; given the absence of isolate-level episode linkage, closely related isolate pairs could not be definitively resolved as separate cases. The remaining 5 patients (29%) had no history of TB in the past. Notably, none of the patients had received Pa, as it was not yet in programmatic use.

Routine pDST data were available for all 9 patients identified as resistant to DLM via REMA testing (i.e., exceeding the resistance cut-off ≥ 0.06 μg/ml). Comprehensive pDST were available for 8 patients, while 1 had limited data, lacking results for first-line drugs. Based on standard NCLH testing protocols, the resistance profile of these 9 patients were as follows: four patients (44.4%) were categorized as extensively drug-resistant (XDR), three (33.3%) as multidrug-resistant (MDR), and one (11.1%) as pre-XDR [26]. For the remaining patient resistance profile was inferred from genomic data and classified as MDR-TB.

## Discussion

Our investigation of nitroimidazole resistance revealed three key findings including 1) nearly half of *Mtb* isolates (47%) deemed resistant to DLM by routine pDST had a low MIC value by REMA testing; 2) among isolates with elevated MICs the most common resistance associated mutations were within the *ddn* gene (67%); 3) high rates (90%) of cross-resistance among DLM and Pa was detected. These findings show key insights into the limitations of the current pDST methods for the novel nitroimidazole drugs and provide additional data to strengthen our understanding of the genotypic-phenotypic relationships for nitroimidazole susceptibility.

The observed discrepancy between routine pDST and REMA for DLM underscores concerns regarding the reliability of pDST methods for novel drugs. This finding is consistent with previous reports [10,27,28] and highlights that pDST methods for DLM/Pa are not yet fully standardized and may lead to inconsistent or inaccurate resistance classification.

During the study period, the initial DLM resistance rate determined by routine MGIT-based pDST was 7.5% (32/425). Following repeated MGIT testing, the confirmed resistance rate decreased to 4.9% (21/425). Given the low prevalence of confirmed DLM resistance, even a small number of false-resistant results can substantially affect the positive predictive value (PPV) of routine pDST. These findings suggest that confirmatory testing may be considered, particularly for initially resistant isolates, to improve the accuracy of DLM resistance classification.

Despite the relatively small sample size of this study, the predominance of previously reported [9,17,20,25] *ddn* W88* mutation in our sample set, when considered with the results from other studies, suggests that the prevalence of this mutation is influenced not only by drug resistance but also by the genetic background of the *Mtb* strains circulating in the population. Our analysis shows that this mutation mainly appears in the L.2.2.1 lineage (Europe_Russia_W148 outbreak), which may be due to the expansion of a successful strain, founder effects in some areas, or past transmission events, rather than new mutations happening independently. These findings can contribute meaningfully to the reclassification of WHO catalogue, where larger number and geographical diversity of *Mtb* isolates are needed to expand this catalogue and enhance its reliability, as was the case for the 2nd edition [20].

The increase in MIC to Pa among DLM-resistant isolates supports a cross-resistance phenomenon, as previously reported [8–10]. In contrast, other studies have observed very little cross-resistance between DLM and Pa, despite their similar mechanisms of action, and have hypothesized intrinsic resistance factors, such as efflux pump activity, may compensate to enable nitroimidazole resistance, and the low observed cross-resistance suggests that DLM and Pa may be recognized by different efflux pumps [29]. Notably, we identified one isolate with a DLM MIC below the critical concentration that still showed a higher Pa MIC value, highlighting the complex nature of resistance mechanisms to these drugs. High rates of cross-resistance to DLM and Pa have important implications for treatment of high drug-resistant patients, as it may preclude the use of the nitroimidazole drugs which are critical to treatment success in drug-resistant disease [30].

Besides the mutations that are classified as having uncertain significance to resistance in WHO mutation catalogue [20], our dataset revealed 4 previously undescribed mutations (G35fs in *ddn* gene, C565W in *fbiC* gene and Q317R and Q279H in *fgd1* gene). Additionally, we identified one isolate with a high MIC to both DLM and Pa which did not exhibit any mutations in known resistance-associated genes. We also found one isolate with MIC below the CC that harbored a mutation in *fbiC*, classified as having uncertain significance in WHO mutation catalogue. These results underscore that our knowledge regarding the genetic determinants of DLM/Pa resistance is still limited [8,12,27,31].

Our study has several limitations. One of them is small sample size and incomplete epidemiological data. Therefore, we focused our analysis on genes previously described as being associated with DLM/Pa resistance, but it is possible that other genetic determinants of resistance exist in other genomic regions not described yet. Furthermore, because H37Rv MICs were truncated at the lowest concentrations tested, exact MIC endpoints could not be determined, limiting direct comparison with published MGIT MICs. Differences between REMA and MGIT methodologies, including variability in MIC determination and the lack of standardized breakpoints for REMA, may also have contributed to the observed discrepancies and should be considered when interpreting our findings. In addition, during our study period, no pDST methods for Pa had been implemented into routine laboratory practice at NCLH, and we could not incorporate routine pDST data for the latter drug. Finally, the study design did not allow reliable estimation of the sensitivity or specificity of MGIT-based drug susceptibility testing, as only isolates classified as resistant by MGIT were subjected to confirmatory REMA MIC and WGS.

In conclusion, our findings indicate that among DLM-resistant cases in Georgia, mutations in the *ddn* gene predominated and occurred in strains with high DLM MICs. We also observed high rates of DLM/Pa cross-resistance, as well as the divergence between routine pDST and REMA testing results, underscoring the necessity of CC recommended for the phenotypic DST. Expanding these data with larger datasets in the future will improve our ability to detect susceptibility and resistance more precisely and keep novel drugs as effective treatment option for TB.

## Supporting information

S1 Table Accession of sequencing datasets used in this study. (DOCX)
