# Supplementary material for: Associating Mycobacterium tuberculosis genomic variants with minimum inhibitory concentrations to delamanid and pretomanid
Source: PLoS One. 2026 Oct 5;21(10):e0359717. doi: 10.1371/journal.pone.0359717 (PMC13637882; doi:10.1371/journal.pone.0359717)
Supplement: Supplementary file 1 [file pone.0359717.s001.docx]

| G_NUMBER | BIOPROJECT | SAMPLE_ACCESSION | RUN_ACCESSION |
| --- | --- | --- | --- |
| G702633 | PRJEB39561 | SAMEA121975353 | ERR16843674 |
| G702636 | PRJEB74234 | SAMEA115432909 | ERR12916926 |
| G702673 | PRJEB39561 | SAMEA117703570 | ERR15577313 |
| G702690 | PRJEB39561 | SAMEA117703511 | ERR15577237 |
| G702708 | PRJEB39561 | SAMEA114640632 | ERR12307399 |
| G702713 | PRJEB39561 | SAMEA117703524 | ERR15577260 |
| G703226 | PRJEB39561 | SAMEA117703441 | ERR15577165 |
| G703239 | PRJEB39561 | SAMEA117703445 | ERR15577169 |
| G703244 | PRJEB39561 | SAMEA121971427 | ERR16843675 |
| G703262 | PRJEB39561 | SAMEA121971428 | ERR16843676 |
| G703277 | PRJEB39561 | SAMEA117703466 | ERR15577191 |
| G724028 | PRJEB39561 | SAMEA121971429 | ERR16843677 |
| G724056 | PRJEB39561 | SAMEA121971430 | ERR16843678 |
| G724063 | PRJEB39561 | SAMEA117703613 | ERR15577392 |
| G742413 | PRJEB39561 | SAMEA117703525 | ERR15577261 |
| G742988 | PRJEB39561 | SAMEA121971431 | ERR16843679 |
| G748744 | PRJEB39561 | SAMEA121971432 | ERR16843680 |
